# Supplementary figures and images for: Adult hospitalizations from immigration detention in Louisiana and Texas, 2015–2018
Source: PLOS Glob Public Health. 2022 Aug 3;2(8):e0000432. doi: 10.1371/journal.pgph.0000432 (PMC10022120; doi:10.1371/journal.pgph.0000432)

**S1 Fig: Identification of Immigration Detention-related hospitalizations**


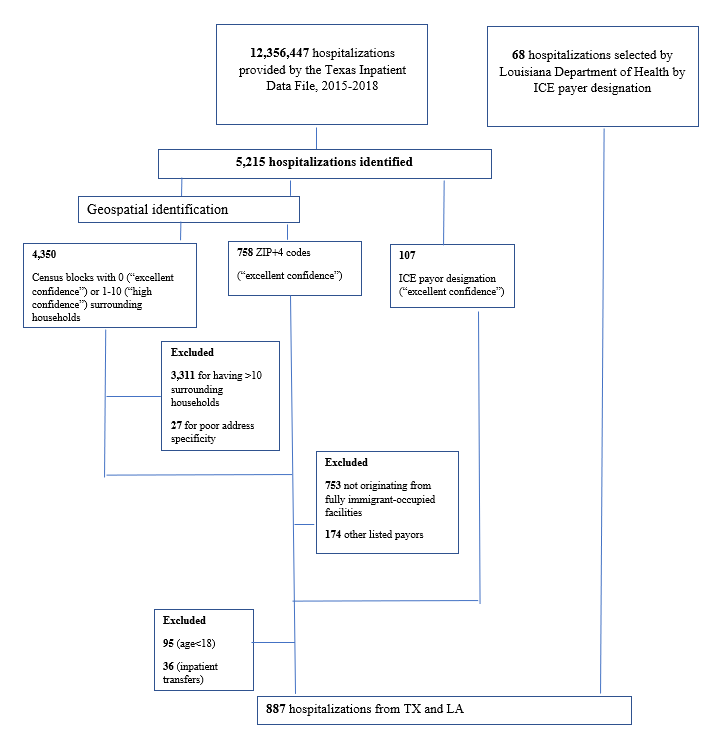

Supplement: S1 Fig — (DOCX) [file pgph.0000432.s001.docx]
