# Supplementary material for: Adult hospitalizations from immigration detention in Louisiana and Texas, 2015–2018
Source: PLOS Glob Public Health. 2022 Aug 3;2(8):e0000432. doi: 10.1371/journal.pgph.0000432 (PMC10022120; doi:10.1371/journal.pgph.0000432)
Supplement: S1 Table — (DOCX) [file pgph.0000432.s002.docx]

**S1 Table: Principal Diagnoses associated with hospitalizations with “excellent confidence” of coming from a detention facility fully occupied by immigrants. ^*,^**^†^

| **Disease Category** | **Count** | **(%)** |
| --- | --- | --- |
| Cardiovascular Disease | 72 | 12.95 |
| Gastrointestinal Disease | 53 | 9.53 |
| Heat exposure, Rhabdomyolysis, and Syncope | 34 | 6.12 |
| Infectious Disease | 160 | 28.8 |
| Neurological Disease | 29 | 5.22 |
| Other | 86 | 15.46 |
| Psychiatric disease | 28 | 5.04 |
| Pulmonary Disease | 23 | 4.14 |
| Renal disease | 40 | 7.2 |
| Trauma and Toxic Exposure | 31 | 5.58 |
| Total | 556 | 100 |

^*^Categories are collapsed for cell sizes greater than 15 for patient privacy in accordance with the Data Use Agreement with Texas Department of State Health Services

^†^“Excellent” confidence refers to hospitalizations linked to ICE’s payor code, linked to an immigration detention center’s ZIP+4 code, or census blocks containing immigration detention facilities and no other residences.
