# Supplementary material for: Adult hospitalizations from immigration detention in Louisiana and Texas, 2015–2018
Source: PLOS Glob Public Health. 2022 Aug 3;2(8):e0000432. doi: 10.1371/journal.pgph.0000432 (PMC10022120; doi:10.1371/journal.pgph.0000432)
Supplement: S2 Table — (DOCX) [file pgph.0000432.s003.docx]

**S2 Table: ICU and intermediate-ICU admissions associated with hospitalizations with “excellent confidence” of coming from a detention facility fully occupied by immigrants. ^*,^**^†^

| **Advanced care level** | **N** | **%** |
| --- | --- | --- |
| No ICU | 376 | 66.79 |
| Step-down only | 131 | 23.56 |
| ICU | 56 | 10.07 |
| Total | 563 | 100 |

^*^Categories are collapsed for cell sizes greater than 15 for patient privacy in accordance with the Data Use Agreement with Texas Department of State Health Services

^†^“Excellent” confidence refers to hospitalizations linked to ICE’s payor code, linked to an immigration detention center’s ZIP+4 code, or census blocks containing immigration detention facilities and no other residences.
