# Supplementary material for: Adult hospitalizations from immigration detention in Louisiana and Texas, 2015–2018
Source: PLOS Glob Public Health. 2022 Aug 3;2(8):e0000432. doi: 10.1371/journal.pgph.0000432 (PMC10022120; doi:10.1371/journal.pgph.0000432)
Supplement: S3 Table — (DOCX) [file pgph.0000432.s004.docx]

**S3 Table: Principal Diagnoses associated with hospitalizations with “good confidence” of coming from a detention facility fully occupied by immigrants. ^*,^**^†^

| **Disease Category** | **Count** | **(%)** |
| --- | --- | --- |
| Cardiovascular Disease | 19 | 6.33 |
| Gastrointestinal Disease | 29 | 9.7 |
| Heat exposure, Rhabdomyolysis, and Syncope | 15 | 5.0 |
| Infectious Disease | 49 | 16.3 |
| Psychiatric disease | 68 | 22.7 |
| Pulmonary Disease | 18 | 6.0 |
| Renal disease | 20 | 6.7 |
| Other | 82 | 27.3 |
| **Total** | **300** | **100** |

^*^Categories are collapsed for cell sizes greater than 15 for patient privacy in accordance with the Data Use Agreement with Texas Department of State Health Services

^†^“Good” confidence refers to hospitalizations linked to census blocks containing immigration detention facilities and between 11 and 25 residences.
