# Supplementary material for: Adult hospitalizations from immigration detention in Louisiana and Texas, 2015–2018
Source: PLOS Glob Public Health. 2022 Aug 3;2(8):e0000432. doi: 10.1371/journal.pgph.0000432 (PMC10022120; doi:10.1371/journal.pgph.0000432)
Supplement: S4 Table — (DOCX) [file pgph.0000432.s005.docx]

**S4 Table: ICU and intermediate-ICU admissions associated with hospitalizations with “good confidence” of coming from a detention facility fully occupied by immigrants. ^*,^** ^†^

| **Admission status** | **N** | % |
| --- | --- | --- |
| No ICU | 228 | 76.3 |
| Intermediate ICU only | 40 | 13.3 |
| ICU | 31 | 10.3 |
| Total | 300 | 100% |

^a^Categories are collapsed for cell sizes greater than 15 for patient privacy in accordance with the Data Use Agreement with Texas Department of State Health Services

^b^ Good confidence refers to hospitalizations linked to census blocks containing immigration detention facilities and between 11 and 25 residences.
