# Supplementary material for: Adult hospitalizations from immigration detention in Louisiana and Texas, 2015–2018
Source: PLOS Glob Public Health. 2022 Aug 3;2(8):e0000432. doi: 10.1371/journal.pgph.0000432 (PMC10022120; doi:10.1371/journal.pgph.0000432)
Supplement: Attachment — Submitted filename: Adult Response to Reviewers (final).docx [file pgph.0000432.s006.docx]

Journal Requirements:

**Comment:** We see that your study includes live participants, but you have not included an Ethics Statement. Please update your manuscript file to include an Ethics Statement subsection to your Materials and Methods section. It should include:
i) The full name(s) of the Institutional Review Board(s) or Ethics Committee(s)
ii) The approval number(s), or a statement that approval was granted by the named board(s) 
iii) (for human participants or donors) - A statement that formal consent was obtained (must state whether verbal/written) OR the reason consent was not obtained (e.g. anonymity)

***Response:*** *An ethics statement has been added as requested.*

**Comment:** In the online submission form, you indicated that "Data is available from the Texas Department of State Health Services and the Louisiana Department of Health.". All PLOS journals now require all data underlying the findings described in their manuscript to be freely available to other researchers, either 1. In a public repository, 2. Within the manuscript itself, or 3. Uploaded as supplementary information.
This policy applies to all data except where public deposition would breach compliance with the protocol approved by your research ethics board. If your data cannot be made publicly available for ethical or legal reasons (e.g., public availability would compromise patient privacy), please explain your reasons by return email and your exemption request will be escalated to the editor for approval. Your exemption request will be handled independently and will not hold up the peer review process, but will need to be resolved should your manuscript be accepted for publication. One of the Editorial team will then be in touch if there are any issues.

***Response:*** *Thank you. Unfortunately, the data we have is protected by Data Use Agreements with the States of Texas and Louisiana, and we are banned from even creating categories with cell sizes less than 15. We will not be able to upload individual level data unfortunately.*

Reviewer's Responses to Questions

**Comments to the Author**

1. Does this manuscript meet PLOS Global Public Health’s [publication criteria](https://journals.plos.org/globalpublichealth/s/criteria-for-publication)? Is the manuscript technically sound, and do the data support the conclusions? The manuscript must describe methodologically and ethically rigorous research with conclusions that are appropriately drawn based on the data presented.

Reviewer #1: Partly

Reviewer #2: Yes

Reviewer #3: Yes

Reviewer #4: Yes

2. Has the statistical analysis been performed appropriately and rigorously?

Reviewer #1: Yes

Reviewer #2: Yes

Reviewer #3: Yes

Reviewer #4: Yes

3. Have the authors made all data underlying the findings in their manuscript fully available (please refer to the Data Availability Statement at the start of the manuscript PDF file)?

The [PLOS Data policy](https://journals.plos.org/globalpublichealth/s/data-availability) requires authors to make all data underlying the findings described in their manuscript fully available without restriction, with rare exception. The data should be provided as part of the manuscript or its supporting information, or deposited to a public repository. For example, in addition to summary statistics, the data points behind means, medians and variance measures should be available. If there are restrictions on publicly sharing data—e.g. participant privacy or use of data from a third party—those must be specified.

Reviewer #1: Yes

Reviewer #2: Yes

Reviewer #3: Yes

Reviewer #4: No

4. Is the manuscript presented in an intelligible fashion and written in standard English?

PLOS Global Public Health does not copyedit accepted manuscripts, so the language in submitted articles must be clear, correct, and unambiguous. Any typographical or grammatical errors should be corrected at revision, so please note any specific errors here.

Reviewer #1: Yes

Reviewer #2: Yes

Reviewer #3: Yes

Reviewer #4: Yes

5. Review Comments to the Author

Please use the space provided to explain your answers to the questions above. You may also include additional comments for the author, including concerns about dual publication, research ethics, or publication ethics. (Please upload your review as an attachment if it exceeds 20,000 characters)

Reviewer #1: This study touches upon one of the most important public health issues, increasing rates of immigrant detention and the consequent health issues in the US. The study very strongly highlights the need for more data in this important area of research.

The authors have described in detail the methodology; however, a couple of concerns need to be addressed.

1. The authors included patients classified as “high confidence” in their primary analysis. This means patients from facilities linked to a census block with 1-10 housing units were included in the analysis. This leads to a greater degree of loss of confidentiality as fewer number of housing units may reveal the patient identity much easily. In this context, it will be interesting to know who (authors or non-authors) were involved in using the satellite images to identify the centers versus houses, and the mechanism used to avoid identifying the houses to protect confidentiality.

**Response:** *Thank you. This might need some clarification on our part. The households count per census block was via US Census data, not by geospatial identification. The only purpose of using geospatial imaging was to confirm that the census block was indeed co-located with the detention center. We did not identify non-detention addresses from geospatial imaging.*

**Comment**: 2. How much change will happen in the overall results, if only “excellent confidence” cases are analyzed? Is it possible to restrict analysis only to this group?

**Response**: *We have included the results to that question in our Supplement. Of note, the ICU admission rate does increase to 33.2%. The distribution of illnesses remains the same, with the except of psychiatric cases, which are spatially concentrated in a* *single site, the South Texas Detention Center, which is only identifiable with the “good confidence” approach.*

Other comments:
1. While the authors’ observation that the severity of illness may be higher than expected may be valid, we need more objective data to substantiate the claim. We do not know the duration stay in the center before these patients were referred to health facilities or diagnosed with a condition or underwent surgery. It may also be that they had underlying conditions which were detected once they reached the center. Therefore statements like “there is a tradeoff between a low willingness for facilities to hospitalize patients and a high severity of illness upon hospital admission” may be avoided/modified.

**Response**: *Thank you for these comments. We have amended the corresponding portion of the discussion to include the concerns included above.*

**Comment**: 2. The very high number of appendicitis and appendicectomy in the group needs more explanation.

**Response**: *Thanks for this observation and question. Data shows that appendicitis makes up around 0.6% of hospitalizations compared to 2.3% of hospitalizations in this sample. However, it is unclear whether this is related to a lower proportion of elective admissions among patients in detention or to increased incidence of appendicitis in the population. Further research is required.*

**Comment**: 3. A further breakup of the 18-45 age groups into smaller groups (at least splitting the very young age groups out) may be useful. This may help to further clarify the high rate of utilization of cardiac care.

**Response**: *Thank you. We broke down age groups further in Table 1, and then provided the average age for advanced care admissions (40) and for cardiac related advanced care admissions (50).*

Reviewer #2:

**Comment:** Clear and important research questions that is properly answered in the study. This is a novel topic that has not been researched previously, and of great importance and implications. There are no fundamental flaws in the design, and has strong technical rigour.

The papers is well-written, clear and easy to read. Tables and figures are clear and simplified. Abstract is strong, clear and summarises the paper clearly. Methods and results are accessible, justified and clearly presented, they support the conclusion.

Minor comments:
Include more detail in methodology on statistical analysis used and justification.

**Response*:*** *First, thanks so much for the kind comments. Our analysis is largely descriptive, which we clarified in the methods section.*

.
**Comment:** Conclusion requires more details on implications of the study results, how and what can be done with the results, e.g. for policymakers, and/or suggestions for future research to compliment this study.

**Response:** *Thank you. We amended our recommendations to policymakers to focus on the need for enhanced psychiatric screening to prevent mentally ill individuals from being placed in detention. We also mentioned the need of more detailed health data paired with detention characteristics (e.g., length of stay in detention), which will help strengthen causal analysis.*

Conclusion

Reviewer #3:

**Comment:** A very nice and well-written quantitative piece on hospitalizations stemming from detention centers in LA and TX. It is an important global health piece that will be an excellent compliment to qualitative explorations and government reports of similar topic.

I have two substantive suggestions:
1. Given the available data from ICE and CBP can the authors provide some information about the detainees in the centers the hospital data is coming from? I assume that they are primarily from Mexico/Northern Triangle countries, but do we have more information (country of origin, language spoken, etc.)?

**Response:** *We unfortunately do not have data on countries of origin in our inpatient dataset, but another dataset, organized by the Transactional Records Access Clearinghouse at Syracuse University, does contain this information from data attained from the federal government. That data has now been included in the results. We have also included data on race and ethnicity.*

**Comment**: 2. The authors should engage more with implications and connections to larger picture of migration in their discussion. What can this data tell us about the conditions from which migrants are leaving and how that impacts morbidities and mortalities in detention. What does ICE and CBP need to be better prepared to deal with? For example, in the paragraph on the burden of psychiatric illnesses (lines 251-259), a sentence or two on the conditions (violence, poverty, etc) that prompt much of the migration would parallel the data and strengthen the argument that ICE & CBP are not doing enough to meet these acute medical needs.

***Response:*** *Thank you, we have referenced pre-migration conditions as suggested.*

Minor suggestions:

**Comment**: Make the relevance of LA and TX earlier in the piece. At first glance it seems like an unlikely selection of states. I would move the statement that 1 in 3 detainees are in TX and LA from parentheses and move it up higher in the introduction.

**Response** *Thank you for that suggestion. We have moved the statement accordingly.*

**Comment**: Footnote 1: the footnote was published in 1999 but is used to reference data from 2019. I tried to visit the link (out of curiosity of the stats) and URL didn't work.

***Response*** *Thanks for the catch! The citation has been corrected.*

Reviewer #4:

**Comment:** This study tabulates hospitalization data of immigration detention centers in Texas (the vast majority of the data) and Louisiana during the years 2015-2018, focusing on fully immigrant-occupied facilities. The authors do not make their curated data set available. I appreciate the effort required to rigorously identify facilities meeting their criteria for being fully immigrant-occupied, while excluding locally sourced hospitalizations. The authors describe in great detail what is more clearly understood in the tables. [As a minor point, the heading the second column in Tables 2 and 3 should be Count.]

While I understand the significance of reporting these data from immigrant-occupied facilities, little can be discerned---beyond conjecture---in the absence of comparative data. I suggest that the authors add hospitalization data from regular state penal institutions; given the minor contribution of the Louisiana data, focus on Texas. From the standpoint of public health, comparisons of the fully immigrant-occupied and regular state penal facilities would better identify clear areas of concern. The authors commented on tuberculosis, psychiatric disease, and skin abscesses as being of particular concern, but in the absence of comparative data it is challenging to call attention to poor facility management. I'm left with the question: Where do these results fall among other studies of hospitalization of prisoner populations.

**Response**: *Thank you for your comment. A study from Texas showed that among prison hospital discharges across the state, 22.4% were related to cardiac related illness and 18% were related to gastrointestinal illness, with the other admissions related to other medical causes. This does not include psychiatric disease since state prisons often triage psychiatric illness to internal psychiatric units. However, emergency department (ED) admission data from Tennessee and New York State show that 4% and 13% of presenting diagnoses were psychiatrically related. This is likely however, due to differences in baseline co-morbidities between immigrant and local populations: Among non-immigrant patients who presented to ED from prisons, 35% have a comorbidity of hypertension, 16% have a comorbidity of coronary artery disease, 11% have a comorbidity of diabetes, and 45% have a comorbidity of psychiatric illness. We have added a paragraph to the discussion raising this issue.*
